# From discovery to production: Scale-out of continuous flow meso reactors

**DOI:** 10.3762/bjoc.5.29

**Published:** 2009-06-09

**Authors:** Peter Styring, Ana I R Parracho

**Affiliations:** 1 Department of Chemical & Process Engineering, The University of Sheffield, Mappin Street, Sheffield S1 3JD, United Kingdom

**Keywords:** catalysis, continuous flow, Kumada reaction, parallel, scale-out

## Abstract

A continuous flow parallel reactor system has been developed to provide a rapid and seamless transition from the discovery phase and production phase of chemical synthesis, particularly in low volume-high value pharmaceuticals production. Using a single fixed bed catalytic meso reactor, reactions can be screened on a small discovery scale over short time scales. The intensified process produces sufficient material for a full analysis. By replication of the single reactor in parallel, the same chemistry can be achieved on a larger scale, on a small footprint and without the mass and heat transport limitations of reactor scale-out in batch.

## Introduction

Cross-coupling reactions are an essential tool in organic synthesis, from pharmaceuticals through to functional materials. In the majority of cases on the laboratory scale, coupling reactions are performed in stirred batch reactors such as round-bottom flasks using homogeneous catalysts and often activating agents such as substituted phosphines, or else catalysts possessing ligands with complex and expensive motifs [1]. Often, the scale at which such reactions are performed is far greater than that required for initial characterisation and property screening [2–3]. Once a compound is identified for further study and even commercialisation it is then required to be produced on a larger scale. The problem is that to move from small discovery to larger pilot or commercial scale production is not simply a case of scaling up the quantities of reagents and solvents [4]. There are also supply issues when dealing with large scale syntheses and at times it may be economically sensible to move a reactor to the supply source rather than vice versa, particularly where hazardous chemical feedstocks are involved [5]. Furthermore, small footprint processes would be advantageous for safety reasons, particularly containment in the case of a failure [2–3]. Scaling up in volume of a reactor has implications on the mass and heat transfer within the system so the reaction must be re-optimised for the prevailing conditions. This is a time-consuming process and often results in the abandoning of the bench scale process in favour of conditions more favourable in bulk. In particular, the use of homogeneous catalysts in scaled-up processes is problematic. Homogeneous catalysts often decompose or dissociate under reaction conditions so that the true active catalyst is not in fact the compound added at the onset of the reaction. This makes catalyst recovery and re-cycling problematic if not impossible. It also leads to the possibility of metal contamination at trace levels in the final product. Furthermore, added phosphines and other activating agents tend to be expensive and also difficult to recover, adding both an environmental and economic burden on the process.

**Scheme 1:**
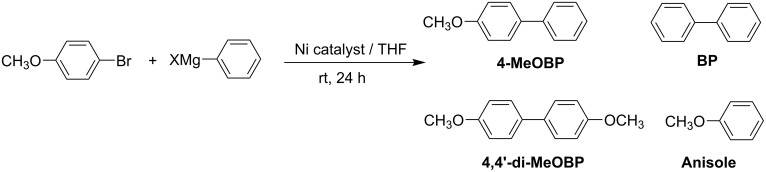
Synthesis of 4-methoxybiphenyl (4-MeOBP) and by-products in the Kumada reaction.

Previously, we have described a series of catalysts based on nickel(II) and palladium(II) that have functionalised salen-acac (salenac) ligands that were functionalised such that they could be covalently bound to organic or inorganic polymer supports [6–10]. Such materials show the beneficial properties of homogeneous catalysts (activity and selectivity) as well as those of a heterogeneous catalyst (robust and re-usable). These materials have been termed ‘androgynous’ due to these dichotomous properties [8]. The palladium catalysts are active in the Suzuki and Heck coupling reactions at elevated temperatures while the nickel catalyst is active in the Kumada coupling [11–12] reaction at room temperature in batch (Scheme 1) and continuous flow reactors. In the latter, the catalyst is packed into a 3 mm diameter glass reactor tube of length 25 mm and the reagent solution flowed using a syringe pump [8–9,13]. This is termed as meso reactor as it shows many of the benefits of a micro reactor but at a slightly larger volume. Using a mesoporous version of the catalyst the rate of reaction was enhanced over 3000 times which meant that useful quantities of material could be produced within minutes rather than overnight [13].

In this paper, we report on the fabrication and testing of a new parallel reactor capable of scaling out chemistries using the same chemistries developed on the discovery scale, while retaining a small area footprint that sits on a standard stirrer hot-plate and is capable of being employed in a standard laboratory fume cupboard. Despite a number of publications describing the theory and practicalities of scaled-out micro and meso reactors [14–15], no practical examples of large-scale production have been described.

## Results and Discussion

### Batch Reactor

Batch reactions were performed using a Radley’s Carousel Parallel Synthesiser fitted with a fuzzy logic controller unit giving temperature control of ±0.1 °C. The systems consists of 12 tubes (45 ml) which are fitted with screw-on Teflon caps that are equipped with valves for the introduction of inert or reactive gases and a septa for the introduction of reagents. The 12 reaction tubes sit in two stacked aluminium blocks; the lower fits on a hotplate-stirrer and can be maintained at a constant temperature, while the upper block has circulating water which cools the top of tubes allowing reactions to be performed under reflux conditions if required.

### Single Channel Mini Reactor

**Figure 1:**
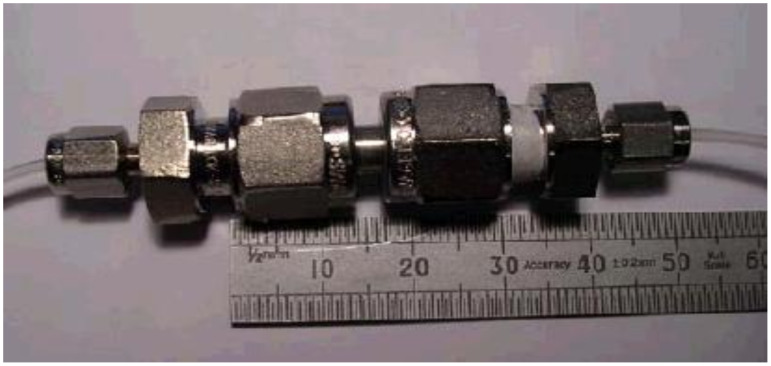
Stainless steel single column flow reactor for discovery scale synthesis.

A stainless steel reactor was constructed using Swagelok components as shown in Figure 1. This has a bed size of 30 mm × 3 mm. The stainless steel column is packed with the catalyst particles; these are held in place by 40 μm woven stainless steel wire mesh with a 25 μm wire integrated in to the reduction unions so that it connects the column to the fluidic delivery system at the reactor entrance and exit in order to prevent loss of the resin catalyst. The reduction unions allowed one end of the reactor to be connected to a disposable solvent-resistant syringe, while the other end was attached to a syringe needle leading to a vessel containing quenching solution. The design of the reactor system makes catalyst filling and removal an extremely easy and quick process. This channel was packed with nickel immobilised onto Merrifield resin (mean average particles size of 45 μm when dry and 72 μm when wet in THF), with approximately 3% nickel loading. A syringe pump (RAZAL A-99) was used to pump a pre-determined volume of the solution of known concentrations at different flow rates.

### Parallel Capillary Reactor

**Figure 2:**
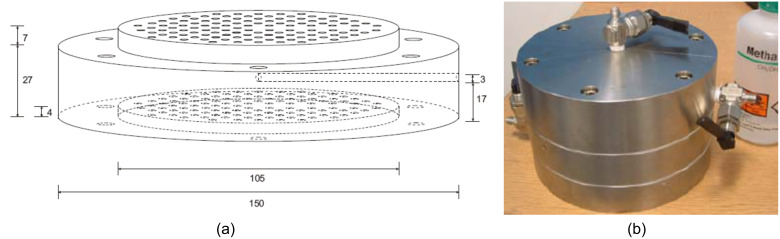
**(a)**: Schematic diagram of the parallel reactor housing (dimensions in mm); **(b)** the stainless steel parallel flow reactor with the reactor housing sat between the flow inlet manifold (bottom) and headspace with gas and liquid outlets (top).

**Figure 3:**
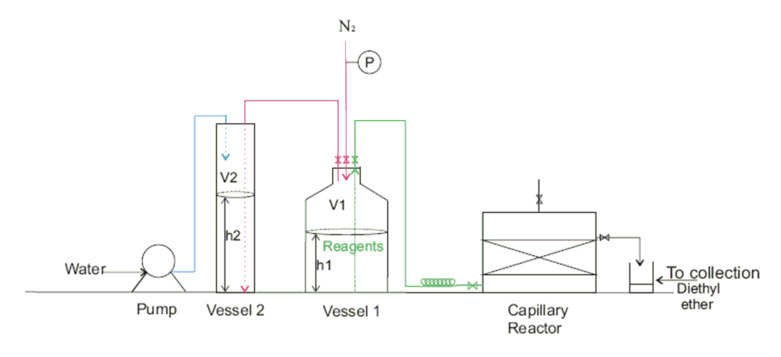
Schematic diagram of the pneumatic pumping system.

The parallel capillary reactor consisted of a capillary block, head space, bottom space inlet manifold, spacer plate, PTFE seal and a packing spacer. Figure 2a shows the schematic of the reactor while Figure 2b is an external photograph of the actual reactor giving an indication of scale. Uniform packing was achieved using an array of stainless steel spacer pins that were inserted into the base of the reactor body while the catalyst was added from the top. The stainless steel mesh was used to hold the catalyst in place at top and bottom of the reactor. Reagents flowed against gravity along the capillary tubes to ensure uniform distribution. The bottom space helps uniform distribution of the reactant solution to be achieved and the reaction products are collected from the head space through a side mounted outlet valve. A gas release valve was included at the top of the headspace unit in order to relieve any pressure build up. The capillary block is the body of the reactor, which contains 120 capillary tubes of 3 mm diameter and 30 mm length. An access point was also drilled in the middle of the reactor, between the reactor tubes, in order to monitor reactor temperature. The inserted temperature probe can be used to control the temperature through feedback with a stirrer hot plate using an IKA Fuzzy Logic controller. Reagents of known concentrations were pumped into the reactor using a pneumatically driven calibrated system with nitrogen as the carrier gas. The pre-mixed reagents were stored in a glass bottle (vessel 1) as shown in Figure 3. In order to maintain a constant flow from the vessel it was necessary to maintain a constant pressure and this was achieved using a second vessel filled with water, which was filled at the same rate as reagents were exiting vessel 1.

**Scheme 2:**
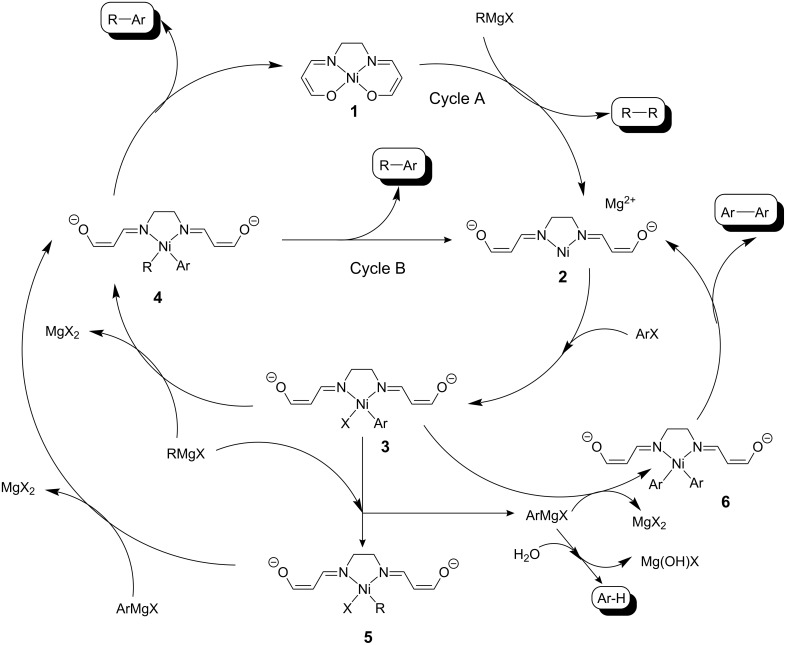
Proposed catalytic cycles for the transformation of 4-haloanisole (Ar-X) and Grignard reagent (RMgX) into 4-methoxybiphenyl (Ar-R) with by-products anisole (Ar-H), biphenyl (R-R) and 4,4′-dimethoxy-biphenyl (Ar-Ar) using the polymer supported nickel catalyst. Ligands are simplified for clarity.

The reaction studied was the Kumada reaction [11–12] which involves the coupling of an aryl halide and a Grignard reagent, in this case 4-bromoanisole and phenylmagnesium chloride, to produce 4-methoxybiphenyl (4-MeOBP, R-Ar). It was also observed that anisole, 4′,4-dimethoxybyphenyl (4,4′-di-MeOBP, Ar-Ar) and biphenyl (BP, R-R) were obtained as reaction by-products. The catalytic cycle that we originally proposed [6] neglects the formation of the other by-products so we investigated the whole scheme in detail. The chloro rather than the bromo Grignard reagent was used, even though it was less reactive, as the waste magnesium chloride or mixed halide was more soluble in THF under the reaction conditions than magnesium bromide and this prevented significant build up of the salt in the reactor while in use, which would otherwise lead to blockage. Batch reactions were performed in order to probe the mechanism for the reaction further. If the activation of the catalyst was important then a pre-wash with the Grignard reagent should improve the yield of the desired cross-coupled product. This was indeed found to be the case. Without any pre-wash or with a 4-bromoanisole pre-wash there was no difference in conversion with a maximum yield of 44% being observed by GC. However, with a pre-wash using phenylmagnesium chloride, the yield increased to 52%. The revised scheme is shown (Scheme 2) and includes the original cycle, shown as cycles A and B. Two mechanisms were originally thought to be important: (a) the activation of the catalyst through sacrificial loss of the Grignard reagent to form the homo-coupled product biphenyl then subsequent cross-coupling to give the desired product (Cycle A); (b) continued production of the cross-coupled product without the need to reactivate the catalyst (Cycle B).

The complex in the free and immobilised form possesses a hard central Ni^2+^ ion chelated by both hard (anionic oxygen) and soft (neutral nitrogen) donor atoms from the ligand [6–7]. Cycle A involves pre-activation of the catalyst by reduction to Ni(0), chelated by soft nitrogen donors, with the production of the homo-coupled organic product through sacrifice of some of the Grignard reagent. This is an essential step as Ni^2+^ is inactive towards the oxidative addition of the organobromide. In general the yield of this homo-coupled product is not proportional to the principal cross-coupled product so the possibility arises that the catalyst takes an alternative pathway (route B) once the catalyst initiation has occurred. As the cross-coupled product is observed as the highest yielding product, it was proposed that the rate constant for route A is smaller than for route B.

The proposed catalytic cycle therefore serves as a working hypothesis for the formation of the principal cross-coupling product, 4-methoxybiphenyl. A study of bromo-Grignard reagent to organo bromide ratio necessary to optimise the yield of cross-coupling product by Phan *et al.* [7–8] showed that the reaction was best performed using a 1:1 substrate ratio with 67% conversion to the desired 4-methoxybiphenyl. The production of biphenyl as a homo-coupling product was also observed in a significant amount of approximately 20% of the final mixture. Indeed, the problem of homo-coupling was encountered in earlier publications involving the coupling reaction under discussion. This by-product can be formed by sacrificial loss of the Grignard reagent in the reduction of Ni(II) to the real catalytically active Ni(0) form as the first step in the proposed catalytic cycle mentioned above. However, the composition of the final reaction mixture also included anisole and 4,4′-dimethoxybiphenyl and the level of biphenyl itself indicates that it is also forming by an alternative pathway. Therefore, the catalytic cycle is more complex than originally proposed. However, using the results from this study together with previously obtained data we have been able to construct a viable alternative route that explains the observed by-products.

Product scrambling occurs through trans-metallation between the intermediate nickel complex **3** formed by oxidative addition of the aryl bromide (Ar-Br), and the Grignard reagent. This results in the formation of the homo-coupled 4,4′-dimethoxybiphenyl by-product. Anisole (Ar-H) is formed by hydrodebromination of the aryl bromide *via* the same oxidative addition intermediate complex **3** due to the presence of traces of water in the system. This occurs both in the batch system, where there is only residual water in the solvent, and in the flow system. Even though water is used to equalise the pressure in the flow system, the reactor is protected from the pumping system using a self-indicating silica gel drying tube that is regularly recharged.

### Mini-Continuous Flow Reactor

Previous work carried out by Styring *et al.* [13] has described the use of a salenac nickel complex immobilised on a polymer support for the Kumada reaction in pressure driven mini flow reactor. In further studies [6–8], a similar nickel resin catalyst [6] and a silica supported nickel catalyst were used [7–8]. The Kumada reaction was carried out in a glass mini flow reactor (Omnifit) and good yields were achieved in a matter of minutes, compared to the conversion obtained in batch over a period of 24 h, albeit on a much smaller scale. The optimum conversion and selectivity was achieved using a flow rate of 13 μl min^−1^, with a 67% yield of 4-methoxybiphenyl being achieved using phenylmagnesium bromide.

**Figure 4:**
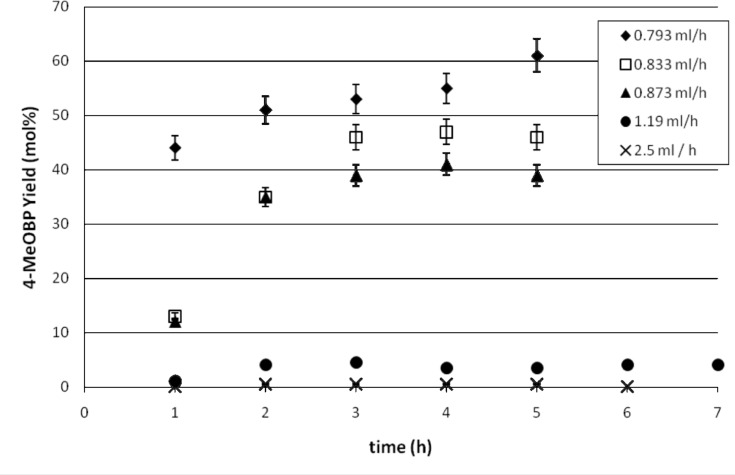
Comparative study of flow rates in a single channel meso flow reactor. The output was sampled hourly and analysed by GC during the period of extended flow.

As the residence time has an important effect on the product yield, and with the aim of scaling out the same reaction in the parallel capillary reactor, the reaction was carried out in the continuous flow reactor at different flow rates. The motivation was to investigate the effect of the residence time of the reagents within the catalyst bed on the coupling process. The mean residence time in a continuous flow reactor is also assumed to be the reaction time. Therefore the reaction was performed at different flow rates in a Swagelok stainless steel column, of the same size of a single channel in the capillary reactor. The reaction was carried out using an equimolar solution of 0.5 M of 4-bromoanisole and phenylmagnesium chloride. The results obtained are shown in Figure 4.

It was observed that as the flow rate increases the yield of 4-methoxybiphenyl decreases, as expected. At higher flow rates of 1.2 and 2.5 ml h^−1^, the yields of 4-methoxybiphenyl were between 0 and 2%. Therefore, it was decided to carry out the Kumada reaction in the capillary parallel reactor at a flow rate of 0.79 ml h^−1^ (13 μl min^−1^) as in previous studies. All conditions were kept the same as in the study of Phan *et al.* [8], however, it was observed that a lower product yield was obtained. This is due to the lower reactivity of the chloro Grignard reagent compared to the bromo derivative used in previous studies. No deterioration of the catalyst was detected over the course of the cycles and ICP-ES studies showed the nickel content in the resin was the same as when the catalyst was initially prepared.

An important point of concern when using heterogeneous catalyst is its lifetime, particularly for industrial and pharmaceutical applications. In an ideal case [16] the catalyst can be recovered and reused several times before it eventually deactivates. At the same time, catalyst recovery can also reduce the environmental pollution caused by heavy metals used in the catalyst systems. When using a supported metal catalyst, another critical issue is the possibility that some active metal migrates from the solid to the liquid phase and this leached metal would become responsible for a significant part of the catalytic activity which would then occur homogeneously. When the solid catalyst was removed from a batch reactor no reaction occurred until the catalyst was replaced, showing leaching is not a problem. The polymer supported catalyst was then tested for its reusability. The catalyst was packed in the Swagelok column and the Kumada reaction was carried out three times without unpacking and without any catalyst regeneration. The conditions were assumed to be the same as in the parallel capillary reactor. In between the reactions the column was flushed with dried THF to remove any excess reagents and salts deposited on the surface of the catalyst. Results showed that the nickel resin catalyst can indeed be re-used in further reactions without a significant degradation in activity. Although there was a slight decrease in activity over subsequent runs, the average yield of 4-methoxybiphenyl still remained greater than 40%.

One of the by products of the Kumada reaction is biphenyl, which is the result of the self-coupling of the phenylmagnesium chloride, as explained in Scheme 2. As the yield of product obtained in the Kumada reaction in this work was comparatively low compared to that of Phan *et al.* [8], the stoichiometry of the reagents was varied accordingly. Two different studies were carried out, in a continuous reaction and the other in a batch process. In the continuous reaction studies, the column was packed with catalyst and pre-washed with a solution of phenylmagnesium chloride in THF (0.5 M) and then rinsed with THF and the experiment was carried out as described previously. Two different stoichiometric ratios (1:1 and 1:1.5 of 4-bromoanisole and phenylmagnesium chloride respectively) were used. There an increase in the 4-methoxybiphenyl yield from 28 to 45% at the higher ratio when a pre-wash was carried out. At the same time the amount of biphenyl produced almost doubled compared to the smaller increase in 4-methoxybiphenyl yield. A comparative study of 1:1 and 1:2 of 4-bromoanisole to phenylmagnesium chloride was then undertaken in a batch process and the production of biphenyl as well as 4,4′-dimethoxybiphenyl was monitored. The nickel complex catalyst instigates the homo-coupling of the Grignard reagent, in this case to produce biphenyl. The yield of 4-methoxybiphenyl was increased by over 20% as seen in Table 1. However, while the yield of 4,4′-dimethoxybiphenyl almost doubled, the yield of biphenyl increased three-fold. If there was simple transmetallation occurring then there should be equity in the yields. However, the elevated value for biphenyl production together with the effectiveness of the Grignard pre-wash supports our theory that the activation of the catalyst through Cycle A is essential for the reaction to proceed effectively. While the comparisons indicates that a stoichiometry of 1:2 yielded better results than a 1:1 stoichiometry, it should be noted that this is both an economic and environmental burden as the excess Grignard reagent is quenched at the end of the process. This could be overcome by building a recycle into the process. In fact, when a mass balance was carried out over the reaction it was found that less Grignard reagent was actually consumed when the higher stoichiometry was used. It should also be noted that the increased stoichiometric ratio has little effect on the turnover frequency (TOF). Although a slight increase was observed, this is within 5% experimental error.

**Table 1:** Comparative study of aryl bromide to Grignard reagent ratio on product and by-product yields.

	Ratio of 4-BA to PhMgCl	
	1:1	1:2

4-Methoxybiphenyl (mol%)	57	80
4-Bromoanisole (mol%)	23	1
Anisole (mol%)	7	9
4,4′-Dimethoxybiphenyl (mol%)	13	23
4-Methoxybiphenyl (mmol)	0.57	0.8
Biphenyl (mmol)	0.03	0.09
TOF (h^−1^)	683	719

### Parallel Flow Reactor

Having established the reaction protocol from the mini-continuous flow reactor runs, and having checked for the reusability of the catalyst, the Kumada reaction was performed in the scaled-out system using the parallel capillary reactor. The stoichiometry used remained constant in all studies, with 1 equivalent of 4-bromoanisole to 1.5 equivalent of phenylmagnesium chloride. This would enhance the yield of product while minimising the Grignard reagent waste in the absence of a recycle stream. Equimolar (0.5 M) solutions were used in all cases.

**Figure 5:**
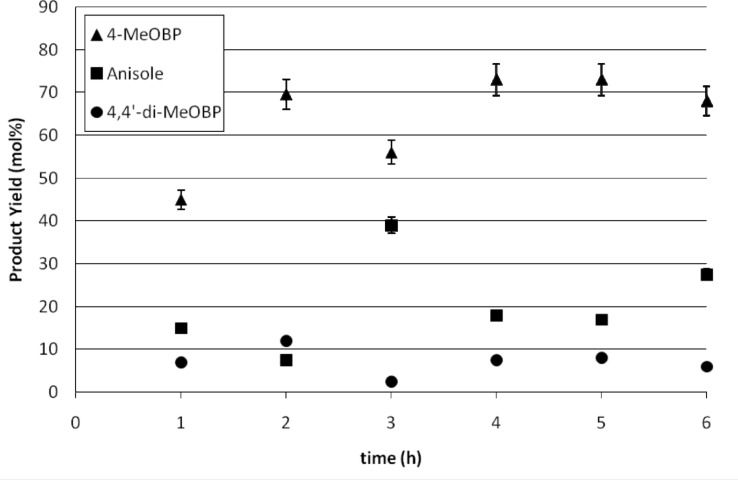
Kumada reaction carried out in a parallel channel meso reactor at a flow rate of 95 ml h^−1^.

In the first study, the reaction was carried out for 6 h at a flow rate of 95 ml h^−1^, which equates to 0.79 ml h^−1^ per channel, as used in the single channel studies. The graph of conversion against time is shown in Figure 5. Samples were taken at hourly intervals by diverting the flow away from the quenching vessel to a separate small volume of quenching solution. The ether layer was decanted off, dried and then analysed by GC. It was observed that the yield increased steadily for the first 3 h and reached a peak of 74% where it remained constant within experimental error. The yield was consistent for a 1:1.5 stoichiometry and higher than the yields originally reported for single channel meso flow reactors. It is proposed that during the first hours of the reaction there is period of induction that conditions the reactor and activates the catalyst. Steady state is then achieved after this induction period, which occurs irrespective of whether or not a pre-wash is used. The induction is therefore most likely to be limited by adsorption of the aryl bromide onto the catalyst surface once catalyst activation is achieved. This is consistent with the Langmuir–Hinshelwood mechanism [17] for surface kinetics which depends on adsorption of both species on to the catalyst surface before reaction can proceed.

**Figure 6:**
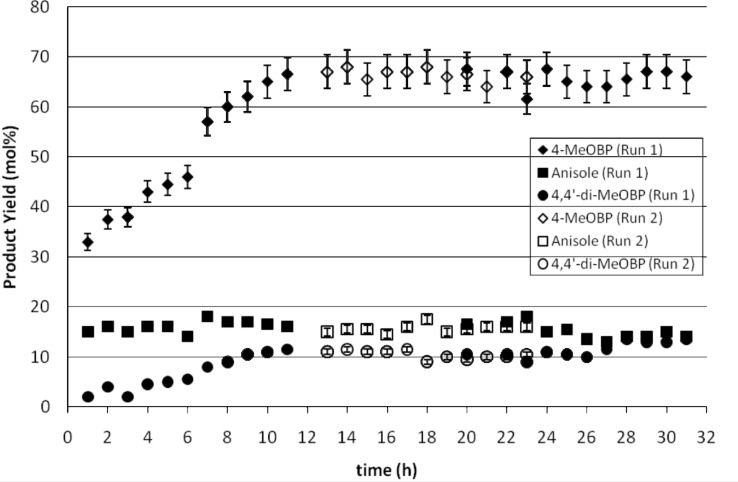
Kumada reaction carried out in a parallel channel meso reactor over a 31-hour period at a flow rate of 95 ml h^−1^ to show catalyst stability and lifetime.

The reaction was then carried out for a period of 31 h using the same flow rate as above, giving a residence time on each of the capillary reactors of 11 min. A linear relationship in the 4-methoxybiphenyl yield with respect to time in the initial hours of the reaction is seen from Figure 6. This induction period is longer than in the six-hour study, however the average yield of 4-methoxybiphenyl after this period was found to be 66% which is consistent with previous studies. As the catalyst used was that used in the six-hour study the possibility of catalyst deactivation was considered. However, the fact that steady state production of around 66% over the study up to 31 h would dispel this theory. Indeed ICP-AES studies showed no significant loss of nickel from the catalyst. The likely scenario is that magnesium salts had become deposited on the catalyst beads on standing between studies and that the initial linear increase in yield was a consequence of the salts being washed off in the continuous flow and adsorption of the organobromide on to the newly freed reactive sites.

It is seen from Figure 6 hat the production of the by-product anisole remains constant throughout the reaction while production of 4-4′-dimethoxybiphenyl increased in a similar way to the main product. The production of 4-methoxybiphenyl during this 31-hour period was found to give an average yield of 58%. This gives a production rate using the parallel capillary reactor of 5.07 g h^−1^ which gives 122 g of 4-methoxybiphenyl per day. If the average at steady state (65%) is taken however, this equates to 5.70 g h^−1^ or 137 g day^−1^. In order to increase production there are two options. One is to replicate further channels in parallel, relying on the fact that the chemistry in each reactor will be identical. The dimensions of the parallel reactor used were such that the design would fit easily onto a standard hotplate-stirrer. However, by making the reactor blocks rectangular they could be readily stacked in three dimensions. The second approach is to increase the flow rate into the reactor, however as this will reduce the residence time it will therefore reduce the effective reaction time with the consequence that the conversion may fall. Therefore, a study in which the flow rate was doubled was undertaken.

**Figure 7:**
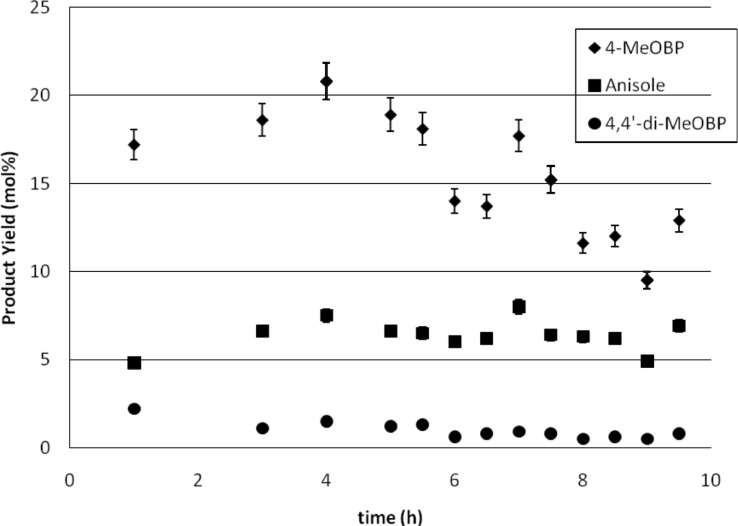
Kumada reaction carried out in a parallel channel meso reactor at a flow rate of 190 ml h^−1^.

A flow rate of 190 ml h^−1^ (1.6 ml h^−1^ per single channel) was used, giving a residence time of 5 min 30 s. The reactor was run for a period of 9.5 h. A decrease in the production yield (%) of 4-methoxybiphenyl with time was observed (Figure 7). The maximum yield was observed after 4 h, however this was only 21%. Yield gradually decreased after this, averaging around 12% although production did not cease. Production of anisole and 4,4′-dimethoxybiphenyl was unaffected however, averaging 6 and 1% respectively, so selectivity decreases with increased flow. Likewise, there was very little change in the turnover frequency (0.35 h^−1^) based on 4-bromoanisole. However, the TOF was significantly reduced from the reaction carried out at half the flow rate (1.5 h^−1^). It should be noted that the TOFs in flow reactors are lower than in batch as we are dealing with small flows through a packed bed reactor and hence the catalyst concentration is necessarily high.

If the rate limiting step in the reaction is the adsorption of the organobromide onto the surface of the catalyst, as we have proposed according to the Langmuir–Hinchelwood mechanism [17], then increasing the flow rate does not give sufficient time for adsorption of the aryl bromide and reaction with the Grignard reagent to occur and hence the conversion will decrease, as was observed. It appears from the data that while adsorption of the aryl bromide can occur there is low availability of the Grignard reagent at high flow rates and hence the homo-coupled product and anisole have the opportunity to form. The product formation decreases as at the start of the reaction there is an induced high concentration of the Grignard reagent on the beads as a result of the pre-wash to activate the catalyst. As the flow proceeds, the concentration is reduced and if it is difficult for more reagent to access the surface then the production slows. This is an interesting observation and we are undertaking detailed mechanistic studies in an attempt to clarify the situation.

Another approach to increasing yield would be to increase the length of the reactor tubes. We standardised on 30 mm channel lengths as we were targeting a generic reactor system that could be used for many different reactions without the need to change components. All our discovery studies were performed in 25 mm glass and 30 mm stainless steel tubes, hence the choice of 30 mm for the parallel reactor. We have not looked at longer discovery reactors because for the Kumada reaction the selectivity is decreased. At equivalent flow rates on longer tubes there is a large increase in the production of biphenyl with increased residence time on the reactor.

## Conclusions

The Kumada reaction was carried out at room temperature, using a nickel catalyst supported on Merrifield resin beads in a Swagelok meso reactor column, which is of the same dimensions as a single channel in a scaled-out parallel capillary reactor. The objective of this research was to demonstrate that it is possible practically to scale-out a reaction carried out on a single channel reactor on a pilot scale/small production reactor by simple replication of the original channel geometry in parallel. This was clearly demonstrated. Reactions optimised in a single channel are simply replicated in a 120-channel reactor. In the case of the Kumada reaction, the yield is up to 137 g over a 24-hour period on a reactor that sits on a standard hot-plate stirrer. Increased yields can be achieved simply by replicating further in two- or even three-dimensions through more sophisticated manifolding.

The catalytic mechanism was studied using batch reactions, and different pre-washes with the starting reagents were carried out. From the results obtained it is proposed that for the nickel salenac complex, the reaction followed a Langmuir–Hinshelwood mechanism, and that a pre-wash with Grignard reagent activates the many reactive sites present on the catalyst. However there can be a probability of other mechanisms occurring and hence further studies are being undertaken.

In the continuous meso flow column, recycling studies of the catalyst were carried out in order to simulate the re-use of the catalyst bed within the capillary parallel reactor. Due to the volume of catalyst needed to pack the reactor and the downtime requirements for repacking it is essential that the catalyst is stable over prolonged periods of operation. This has been shown to be the case and the reactor ran continuously over a 31-hour period with steady state product synthesis without loss of performance.

## Experimental

### Reagents

Chemicals were obtained from Sigma–Aldrich and Fisher. Diethyl ether (99.8%, Fisher), anisole (99%, Aldrich), biphenyl (97%, Aldrich), 4-bromoanisole (99%, Aldrich), phenylmagnesium chloride (2M in THF, Aldrich), mesitylene (98%, Aldrich), 4-methoxybiphenyl (97%, Aldrich), 4,4′-dimethoxybiphenyl (99%, Aldrich), were used as received. THF (99%, Aldrich) was dried over 4 Å molecular sieves.

### Batch Reaction Procedure

Unless otherwise stated, a solution of 4-bromoanisole (0.187 g, 1 mmol) in dry THF (1 ml) was added to a Radley’s Carousel reaction tube containing the required amount of the nickel catalyst. The Grignard reagent, phenylmagnesium chloride (1 M, 1 ml, 1 mmol) in THF was transferred via syringe under a nitrogen atmosphere and added directly into the solution of the organobromide. The mixture was stirred at room temperature for 24 h under a nitrogen atmosphere. To work-up the reaction, saturated aqueous NaCl solution (2 ml) was added to quench excess Grignard reagent. The organic components were extracted into diethyl ether (2 × 3 ml) which was then dried over anhydrous MgSO_4_ and the resulting solution analysed by GC and GC-MS with reference to standard solutions of 4-methoxybiphenyl, anisole and 4,4′-dimethoxybiphenyl.

### Single Channel Mini Reactor Procedure

The reaction of 4-bromoanisole with phenylmagnesium chloride in THF was carried out in the pressure driven mini flow stainless steel reactor. The reactor was filled with the resin beads containing the immobilised nickel catalyst (72 mg). A syringe pump (RAZAL A-99) was used to drive a pre-determined volume of a solution containing equimolar amounts (0.5 M) of the reagents in THF through the reactor at the different flow rates for at least 5 h. No reaction was observed in the mixed solution in the absence of the catalyst. The organic components were extracted into diethyl ether and analysed by GC with reference to standard solutions of 4-bromoanisole.

### Parallel Capillary Reactor Packing Procedure

Each channel of the parallel reactor was filled simultaneously with resin to a preset volume. Uniformity was achieved using a jig constructed of a series of pillars with spacings identical to the layout of the channel positions, each pillar having the same diameter as the internal diameter of the channel. A series of jigs were constructed with different pillar heights to allow different packing volumes to be achieved. The reactor body was placed over the jig and resin beads added to the top of the reactor and spread so that they evenly occupied the channels. The stainless steel mesh was then located on top of the channel assembly and the reactor lid bolted in place. The reactor was then inverted and the jig carefully removed. The second stainless steel mesh was placed over the reactor assembly and the reactor base bolted in place. The reactor was then ready for connection to the pumping and collection pipe work. An average packing of 75.3 g per channel was calculated. It is not possible to isolate individual channels to inspect their contents due to the large number of capillaries present. However, in order to ensure that the packing procedure was consistent we ran tests on a reactor packed with Merrifield resin beads without catalyst attached by pumping a solution of guaiazulene dye in THF (0.5 M) at a flow rate of 95 ml h^−1^ and the reactor top removed. The reactor was inspected visually for the emergence of the blue solution. This was found to occur simultaneously over all 120 channel within a 1 s time range, confirming equal packing over all channels. When all the beads were removed from the reactor, homogeneous staining was observed.

### Parallel Capillary Reactor Procedure

The Kumada reaction between 4-bromoanisole and phenylmagnesium chloride in THF was scaled out in the parallel capillary reactor. The reactor was packed with approximately 8.7 g of nickel complex immobilised on Merrifield resin. This gives an average of 75.3 mg of resin per channel. A mixture of 4-bromoanisole and PhMgCl was placed in a sealed glass bottle in THF under an atmosphere of nitrogen. In each case 4-bromoanisole was the limiting reagent. The mixture was pumped continuously through the nickel resin bed packed in the reactor, using the pressure driven pumping system described previously, at room temperature. The flow rates used were of 95 ml h^−1^ and 190 ml h^−1^, with samples collected at 1-hour intervals during a period of 7 h or more. The organic components were extracted into diethyl ether and analysed by GC with reference to standard solutions of 4-bromoanisole and mesitylene as an internal standard.

### Analysis Methods

A Varian-GC 3900 gas chromatograph fitted with a 15 m column of 0.25 millimetre diameter and 0.25 μm thickness CP-SIL5CB coating. The temperature program was an initial hold at 50 °C for 30 seconds followed by a ramp from 40–110 °C at 40 °C min^−1^ followed by a ramp of 110–250 °C at 20 °C min^−1^. GC-MS analyses were performed using a Perkin Elmer GC-MS with a 30 m × 0.25 mm × 0.25 μm Phenomenex-2B5 column. The temperature program was 60–260 °C at 10 °C min^−1^ with a final temperature isothermal hold for 10 min. The MS limit was set between 50 and 450 Da.
